# Photosensitizer-driven nanoassemblies of homodimeric prodrug for self-enhancing activation and synergistic chemo-photodynamic therapy

**DOI:** 10.7150/thno.59065

**Published:** 2021-04-07

**Authors:** Shenwu Zhang, Ziyue Wang, Zhiqiang Kong, Yuequan Wang, Xuanbo Zhang, Bingjun Sun, Haotian Zhang, Qiming Kan, Zhonggui He, Cong Luo, Jin Sun

**Affiliations:** 1 Department of Pharmaceutics, Wuya College of Innovation, Shenyang Pharmaceutical University, Shenyang, Liaoning, 110016, P. R. China.; 2 Department of Pharmacology, School of Life Science and Biopharmaceutics, Shenyang Pharmaceutical University, Shenyang, Liaoning 110016, PR China.

**Keywords:** Cabazitaxel homodimer, Pyropheophorbide a, Nanoassemblies, Self-enhancing activation, Chemo-photodynamic therapy.

## Abstract

Carrier-free prodrug-nanoassemblies have emerged as promising nanomedicines. In particular, the self-assembled nanoparticles (NPs) composed of homodimeric prodrugs with ultrahigh drug loading have attracted broad attention. However, most homodimeric prodrugs show poor self-assembly ability due to their symmetric structures. Herein, we developed photosensitizer-driven nanoassemblies of homodimeric prodrug for self-enhancing activation and chemo-photodynamic synergistic therapy.

**Methods:** In this work, a pyropheophorbide a (PPa)-driven nanoassemblies of an oxidation-responsive cabazitaxel homodimer (CTX-S-CTX) was fabricated (pCTX-S-CTX/PPa NPs). The assembly mechanisms, aggregation-caused quenching (ACQ) effect alleviation, singlet oxygen generation, self-enhancing prodrug activation, cellular uptake, intracellular reactive oxygen species (ROS) generation and synergistic cytotoxicity of pCTX-S-CTX/PPa NPs were investigated *in vitro*. Moreover, the pharmacokinetics, *ex vivo* biodistribution and *in vivo* therapeutic efficacy of pCTX-S-CTX/PPa NPs were studied in mice bearing 4T1 tumor.

**Results:** Interestingly, PPa was found to drive the assembly of CTX-S-CTX, which cannot self-assemble into stable NPs alone. Multiple intermolecular forces were found to be involved in the assembly process. Notably, the nanostructure was destroyed in the presence of endogenous ROS, significantly relieving the ACQ effect of PPa. In turn, ROS generated by PPa under laser irradiation together with the endogenous ROS synergistically promoted prodrug activation. As expected, the nanoassemblies demonstrated potent antitumor activity in a 4T1 breast cancer BALB/c mice xenograft model.

**Conclusion:** Our findings offer a simple strategy to facilitate the assembly of homodimeric prodrugs and provide an efficient nanoplatform for chemo-photodynamic therapy.

## Introduction

Nowadays, malignant tumors constantly threaten human health 1. Although a variety of emerging therapies have been developed for cancer treatment, chemotherapy remains one of the most common treatments in clinical practice 2. However, the clinical chemotherapy outcomes are usually far from satisfactory, owing to the poor physicochemical properties and tumor targeting capacities of most conventional chemotherapeutic drugs 3. In addition, due to their narrow therapeutic windows, chemotherapeutic drugs usually show severe systemic toxicities 3. Recently, nanotechnology has been widely utilized to address the poor physicochemical properties and off-target effects of chemotherapeutic drugs 4-6. A variety of nanomedicines have been developed and applied for cancer therapy, such as albumin-bound paclitaxel (Abraxane) 7. Notably, noncovalent encapsulation of drugs in conventional nanocarriers has some disadvantages such as low drug loading capacity, potential drug leakage and carrier-related toxicities 8-10. Moreover, complicated preparation techniques have been widely accepted as one of the major obstacles hindering the successful clinical translation of nanomedicines 8-10. To address these challenges, great efforts have been devoted to the construction of simple and efficient nanoparticulate drug delivery systems (nano-DDS).

In recent decades, carrier-free nanomedicines self-assembled from prodrugs have been developed 11-24. Prodrug nanoassemblies have distinct advantages including facile fabrication, good reproducibility, high drug loading capacity, and negligible carrier material-induced toxicities 11-24. In particular, the self-assembled nanoparticles (NPs) of homodimeric prodrugs have attracted considerable attention as a unique and promising nano-DDS 25-27. Homodimeric prodrug-nanoassemblies not only share the same advantages with prodrug-based NPs, but also show much higher drug loading capacities than monomer prodrug nanoassemblies 25-27. However, most homodimeric prodrugs with symmetric molecular structures usually have poor self-assembly ability 25. Therefore, there is an urgent need to tackle the assembly challenge of homodimeric prodrugs.

Photodynamic therapy (PDT) is a clinically approved non-invasive treatment modality with high therapeutic selectivity and low systemic toxicity 28-30. During PDT, tumor cells are killed by reactive oxygen species (ROS) generated by photosensitizers (PSs) under laser irradiation 28-30. Notably, PSs with highly conjugated aromatic structures usually show strong intermolecular interactions 31-33. Thus, we hypothesized that PSs might not only drive the stable assembly of homodimeric prodrugs, but also have the potential to fabricate a simple and versatile co-delivery nanosystem for chemo-photodynamic synergistic therapy. The hydrophobic PSs are expected to endow the nanosystem with strong intermolecular hydrophobic force, hydrogen bonding force, as well as π-π stacking interaction 33. Moreover, the combination of chemotherapy and PDT has been identified as an effective strategy to improve therapeutic efficacy 34-37. More importantly, chemotherapy-associated toxicities and side effects can be significantly alleviated by dose reduction 34-37.

**Figure 1:**
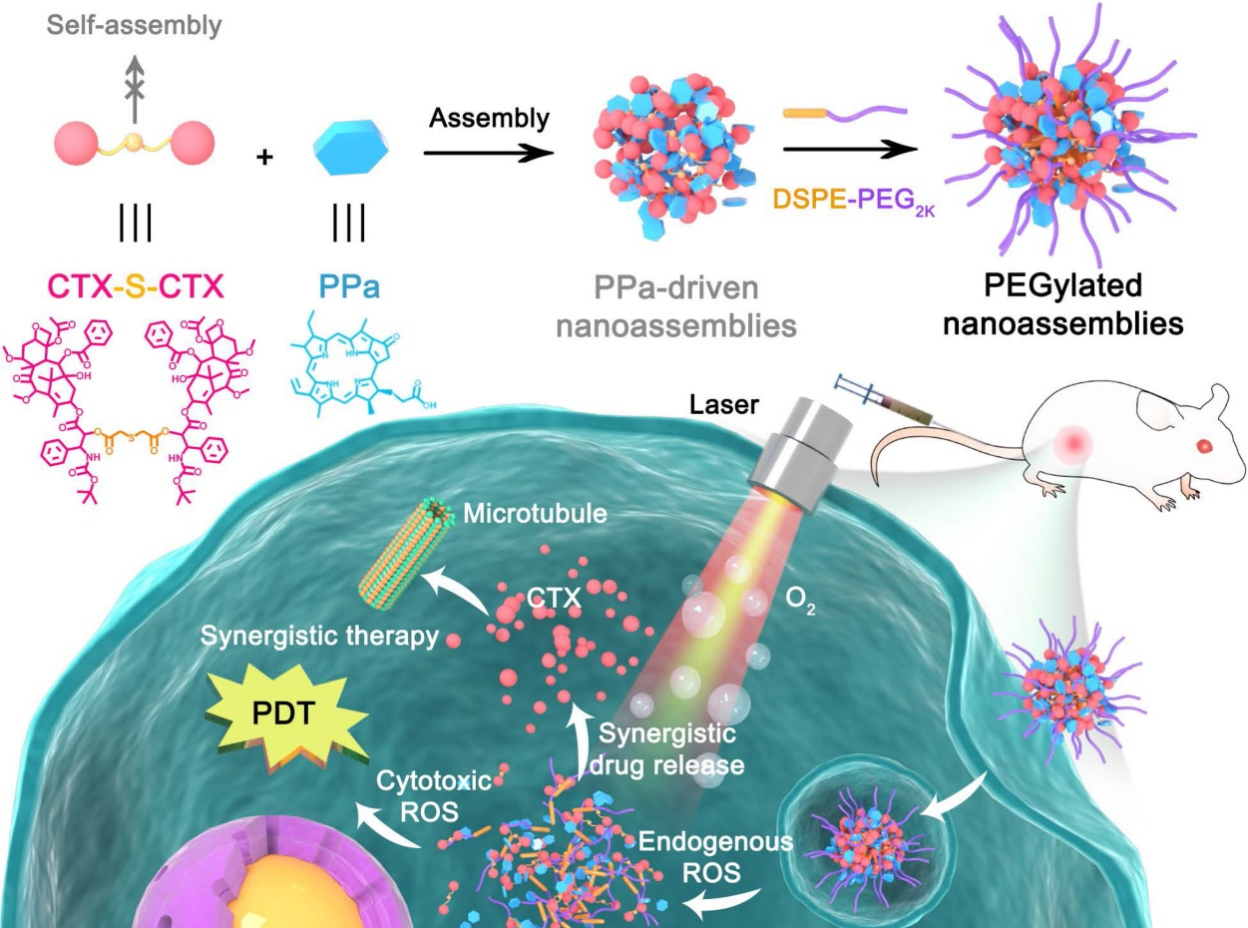
Schematic representation of the PPa-driven nanoassemblies of CTX-S-CTX for self-enhancing chemo-photodynamic therapy. PEGylated NPs were constructed from CTX-S-CTX, PPa and DSPE-PEG_2K_. After internalization into tumor cells, synergistic chemo-photodynamic therapy could be realized with laser irradiation.

To test our hypothesis, we designed a novel ROS-responsive homodimer of two cabazitaxel (CTX) conjugated by a single thioether bond (CTX-S-CTX). Like most homodimeric prodrugs, CTX-S-CTX could not self-assemble into NPs, due to its highly symmetric structure (Figure 1). In the presence of pyropheophorbide a (PPa), CTX-S-CTX facilely assembled into uniform NPs in water. Multiple intermolecular forces were found to be involved in the assembly process, including hydrogen bond, hydrophobic interaction, π-π stacking and π-cation interaction. Notably, the dose ratios of PPa and CTX-S-CTX in the PPa-driven nanoassemblies could be flexibly adjusted to achieve synergistic therapeutic effects. To extend the blood circulation time of NPs, a very small amount (20%, w/w) of phospholipid-polymer conjugate (DSPE-PEG_2K_) was added to the nanoassemblies. The PEGylated NPs (pCTX-S-CTX/PPa NPs) demonstrated multiple therapeutic advantages, including high drug co-loading efficiency, good colloidal stability, long systemic circulation, and strong tumor accumulation. Furthermore, the nanostructure of pCTX-S-CTX/PPa NPs was disassembled by endogenous ROS-triggered activation of CTX-S-CTX, significantly relieving aggregation caused quenching (ACQ) of PPa photoactivity. More importantly, the ROS generated by PPa under laser irradiation together with the intracellular endogenous ROS synergistically facilitated the activation of CTX-S-CTX. As a result, the released CTX and the PPa-generated ROS synergistically delivered potent chemo-photodynamic therapy in a xenograft 4T1 breast cancer mouse model.

## Experimental Section

### Materials

CTX, thioglycolic anhydride (TA), singlet oxygen sensor green (SOSG) kit and ROS assay kit (DCFH-DA) were purchased from Dalian Meilun Biotech Co., Ltd. PPa was obtained from Shanghai Xianhui Pharmaceutical Co., Ltd. 1,2-distearoyl-*sn*-glycero-3-phosphoethanolamine-*N*-[amino(polyethyleneglycol)-2000] (DSPE-PEG_2K_) was obtained from Shanghai Advanced Vehicle Technology Co. Ltd. Cell culture consumables were provided by Wuxi NEST Biotechnology Co., Ltd. Other reagents and solvents used were analytical grade.

### Synthesis of CTX-S-CTX

CTX-S-CTX was synthesized by a two-step esterification reaction. First, TA (2 mmol), DMAP (0.5 mmol), HOBt (4 mmol), and EDCI (4 mmol) were dissolved in 20 mL of dichloromethane, and stirred in an ice bath for 2 h. Then, CTX (2 mmol) was added to the reaction solution and stirred continuously at room temperature for 24 h. The reaction process was monitored by thin-layer chromatography. The intermediate product was separated and purified by preparative liquid chromatography with acetonitrile and water (80:20) as the mobile phase. Similarly, the intermediate product, DMAP, EDCI, and HOBt were mixed in chloromethane, and then CTX was added to the reaction solution after activation in an ice bath. Subsequently, the end product (CTX-S-CTX) was purified by preparative liquid chromatography with acetonitrile as the mobile phase. Finally, successful synthesis of CTX-S-CTX was confirmed by mass spectrometry (MS) and nuclear magnetic resonance (NMR). CTX-S-CTX was dissolved in methanol (20 μg/mL) for MS. The positive ion mass spectrum (+MS) of CTX-S-CTX was determined by high-performance liquid chromatography (HPLC)-MS with the following conditions: mobile phase, methanol and water (80:20); flow rate, 0.2 mL/min; MS detector: Shimadzu LCMS-8060. CTX-S-CTX was dissolved in DMSO-d_6_ (10 mg/kg) for NMR analysis. The ^1^H-NMR of CTX-S-CTX was measured using a Bruker ARX-400 NMR spectrometer with tetramethylsilane as the internal standard.

### Preparation and characterization of PPa-driven nanoassemblies of CTX-S-CTX

Nanoassemblies of PPa and CTX-S-CTX were prepared by a one-step nanoprecipitation method. Briefly, CTX-S-CTX and PPa were dissolved in a mixed solution of absolute ethyl alcohol and tetrahydrofuran (1:1). Then, the solution was added dropwise to purified water and stirred at 1200 rpm for 2 min. Finally, the organic solvent was removed by rotary evaporation to obtain the nanoassemblies (CTX-S-CTX/PPa NPs). PEGylated nanoassemblies (pCTX-S-CTX/PPa NPs) were prepared using the same method but with DSPE-PEG_2K_ (20%, w/w) included with PPa and CTX-S-CTX. The drug loading (DL) efficiency was calculated by the following equations: *DL_non-PEGyalted_*_CTX-S-CTX/PPa NPs_ = (*a*×*M*_PPa_ + *b*×*M*_CTX_) / (*a*×*M*_PPa_ + *b*×*M*_CTX-S-CTX_) × 100%; *DL*_pCTX-S-CTX/PPa NPs_ = (*a*×*M*_PPa_ + *b*×*M*_CTX_) / [1.2 × (*a*×*M*_PPa_ + *b*×*M*_CTX-S-CTX_)] × 100%. *M*_PPa_ is the molecular weight of PPa, *M*_CTX_ is the molecular weight of CTX, *M*_CTX-S-CTX_ is the molecular weight of CTX-S-CTX, and *a* and *b* are the molar ratios of PPa and CTX-S-CTX.

To identify the optimal dose ratio of CTX-S-CTX and PPa, both the particle size and combination index (CI) were used to screen formualtions. The particle sizes of non-PEGylated CTX-S-CTX/PPa NPs at various dose ratios were measured by dynamic light scattering (DLS) (Zetasizer Nano ZS, Malvern). The CIs of CTX solution and PPa solution in various proportions were measured in 4T1 cells. Cells (1 × 10^3^ cells/well) were seeded in 96-well plates and cultured for 24 h. The culture medium was replaced with fresh medium containing CTX solution, PPa solution, or both. After 4 h of incubation, PPa solution and mixed solution of CTX and PPa were exposed to laser irradiation (660 nm, 60 mW/cm^2^, 5 min) and further incubated for 44 h. After incubation, 20 μL of MTT (5 mg/mL) was added to the medium and incubated for 4 h. The medium was then removed and replaced with 200 μL of DMSO, and the absorbance of the produced formazan was measured using a microplate reader (Thermo Scientific, USA). The CI was calculated using the following formula: *CI* = *X*×*AB*_combination_/*A*_alone_ + *Y*×*AB*_combination_/*B*_alone_. *X* is the dose proportion of CTX in the mixture solution of PPa and CTX, *Y* is the dose proportion of PPa in the mixture solution of PPa and CTX, *A_alone_* is the IC_50_ of CTX solution, *B_alone_* is the IC_50_ of PPa solution, and *AB_combiantion_* is the IC_50_ of mixture solution.

After identifying the optimal dose ratio, the particle sizes and zeta potentials of non-PEGylated CTX-S-CTX/PPa NPs and pCTX-S-CTX/PPa NPs were measured. To observe the particle morphology, the NPs were stained with phosphotungstic acid and imaged by transmission electron microscopy (TEM) (HT7700, Hitachi). Salting out is a common phenomenon observed for non-PEGylated NPs. To analyze the effect of DSPE-PEG_2k_ on particle stability, non-PEGylated CTX-S-CTX/PPa NPs or pCTX-S-CTX/PPa NPs were added to phosphate-buffered saline (PBS) (pH 7.4) and morphological changes of the NPs were observed. Additionally, the stability of pCTX-S-CTX/PPa NPs in PBS containing 10% fetal bovine serum (FBS) was investigated by measuring the change in particle size at predetermined time points by DLS.

### PPa-driven assembly mechanisms

The PPa-driven assembly mechanisms of CTX-S-CTX were explored using computer simulations. The Vina protocol in Yinfo Cloud Platform was applied to evaluate the performance of molecular docking 38. Three-dimensional structures of PPa and CTX-S-CTX were calculated with energy minimization in the MMFF94 force field 38. Semi-flexible docking between PPa and CTX-S-CTX was accomplished via AutoDock Vina program, and internal clustering poses and affinity energy were predicted 38. To further identify the interactions and forces in the assembly process, non-PEGylated CTX-S-CTX/PPa NPs or pCTX-S-CTX/PPa NPs were incubated in release medium containing urea, KCl, NaCl, and sodium dodecyl sulfate (SDS) for 12 h at 37 °C 39-40. Changes of non-PEGylated CTX-S-CTX/PPa NPs or pCTX-S-CTX/PPa NPs in the particle size were measured by DLS. Moreover, Gromacs 2019.5 software package was used for molecular dynamics simulations 41. The specific process was as follows: (i) 5000 steps steepest descent method combined with 5000 steps conjugate gradient method was used to optimize the system to avoid unreasonable contact; (ii) the isothermal-isobaric ensemble was used to pre-balance the system, V-Resale temperature coupling and Parrinello-Rahman pressure coupling were used to maintain the temperature at 298 K, the trunking radius of non-bonding was 1.0 nm, and the integration step size was 1 fs; (iii) after balancing, the system was switched to Berendsen's method. The bond length and bond angle were constrained by the Lincs algorithm. The double intercept was 1.0 nm, and Van der Waals interaction was adopted. Long-distance electrostatic interactions were set by Particle-Mesh Ewald method. The system saved a corresponding track file every 10.0 ps. The results were plotted using VMD1.9.3 42.

### Ultraviolet absorption and fluorescence spectroscopy

The ultraviolet (UV) absorption spectra of PPa solution, non-PEGylated CTX-S-CTX/PPa NPs and pCTX-S-CTX/PPa NPs (1 μM, PPa equivalent) were collected from 300 to 800 nm using a microplate reader. The fluorescence spectra of PPa solution, non-PEGylated CTX-S-CTX/PPa NPs, and pCTX-S-CTX/PPa NPs (1 μM, PPa equivalent) were collected from 500 to 900 nm with excitation at 415 nm using a microplate reader.

### Oxidation-sensitive alleviation of the ACQ effect

Briefly, pCTX-S-CTX/PPa NPs (1 μM, PPa equivalent) were incubated in release medium (PBS containing 30% anhydrous ethanol, pH 7.4) containing various concentrations of hydrogen peroxide (H_2_O_2_) at 37 °C for 0, 1, 2, or 4 h. First, changes in the particle size were measured by DLS. Then, the fluorescence spectra of the incubated solutions were measured using a microplate reader with excitation at 415 nm.

### *In vitro* generation of singlet oxygen (^1^O_2_)

**^1^O_2_** generated by free PPa solution or pCTX-S-CTX/PPa NPs was detected using SOSG. In order to explore whether ROS generation was improved by CTX-S-CTX activation and subsequent disassembly of pCTX-S-CTX/PPa NPs, ^1^O_2_ generation was measured following incubation with H_2_O_2_. PPa solution or pCTX-S-CTX/PPa NPs (1 μM, final concentration) were mixed with release medium containing various concentrations of H_2_O_2_. After incubation for various periods, SOSG (1 μM, final concentration) was added to the release medium and the samples were exposed to laser irradiation (660 nm, 50 mW/cm^2^, 5 min). The fluorescence intensity of SOSG was measured using a microplate reader.

### *In vitro* drug release

The* in vitro* release of CTX from pCTX-S-CTX/PPa NPs was investigated in release media under various conditions (H_2_O_2_ or laser). Moreover, PBS (pH 7.4) containing 30% anhydrous ethanol was used as the release medium for studying the* in vitro* release profile of pCTX-S-CTX/PPa NPs. Briefly, pCTX-S-CTX/PPa NPs were added to the release medium and incubated in thermostatic oscillator at 37 ℃. At predetermined intervals, 1 mL of release medium was removed and the concentration of CTX was measured by HPLC.

### Cell culture

Human normal liver cells (LO2) and mouse breast cancer cells (4T1) were cultured in RPMI 1640 medium supplemented with 10% FBS and penicillin and streptomycin. Human epidermoid carcinoma cells (KB) were cultured in DMEM medium supplemented with 10% FBS and penicillin and streptomycin.

### Cellular uptake

The cellular uptake efficiencies of PPa solution and pCTX-S-CTX/PPa NPs were studied in 4T1 cells. Cells (10^5^ cells/well) were seeded in 12-well plates and cultured. The culture medium was replaced with fresh medium containing PPa solution or pCTX-S-CTX/PPa NPs and cultured for 0.5, 2 or 4 h. At these timepoints, the cells were washed and fixed with 4% cell tissue fixator for 15 min, then washed, stained with Hoechst for 10 min, and washed again. Then, the samples were imaged by a laser confocal microscopy (CLSM) (C2, Nikon).

In addition, ultrasonic crushing and protein precipitation extraction methods were used to quantitatively measure cellular uptake and eliminate interference from ACQ. After drug incubation, the cells were digested, suspended, and broken up by ultrasound. Then, PPa was extracted by a methanol protein precipitation method and measured using a microplate reader.

### Cellular ROS generation

4T1 cells (5 × 10^4^ cells/well) were seeded in 24-well plates and cultured for 24 h. Then, the culture medium was replaced with fresh medium containing PPa or pCTX-S-CTX/PPa NPs (50 nM, PPa equivalent) and incubated for 2 or 4 h. After incubation, the drug-containing medium was replaced with fresh medium containing DCFH-DA (50 μM) and further incubated for 30 min. Then, the cells were exposed to a laser irradiation (660 nm, 58 mW/cm^2^, 5 min). Fluorescence from DCF was evaluated using an inverted fluorescence microscope (Nikon, Japan).

### Cytotoxicity

The *in vitro* cytotoxicity of pCTX-S-CTX/PPa NPs was studied in 4T1 cells and KB cells using MTT assay under dark conditions. Tumor cells (1 × 10^3^ cells/well) were seeded in 96-well plates and cultured for 24 h. Then, the culture medium was replaced with fresh drug-containing medium (PPa solution, CTX solution, pCTX-S-CTX/PPa NPs, or mixture of CTX and PPa) and incubated for 48 or 72 h. After incubation, 20 μL of MTT (5 mg/mL) was added to the medium and incubated for 4 h. The medium was then removed and replaced with 200 μL of DMSO, and the absorbance of the produced formazan was measured using a microplate reader. The chemo-photodynamic cytotoxicity of pCTX-S-CTX/PPa NPs was evaluated following a similar process. After 4 h incubation with drugs, the cells were exposed to laser irradiation (660 nm 60 mW/cm^2^, 5 min) and further incubated for 44 or 68 h. To investigate the selectivity and safety of pCTX-S-CTX/PPa NPs, the MTT assay was performed in human normal liver cells (LO2) following 48 h drug incubation.

### Animal models

All animal experiments were performed according to the Guide for Care and Use of Laboratory Animals of Shenyang Pharmaceutical University. Sprague-Dawley rats were acquired from Liaoning Changsheng Biotechnology Co. Ltd. BALB/c mice were acquired from Liaoning Changsheng Biotechnology Co. Ltd. A xenograft breast cancer model was established by injecting 100 μL of 4T1 cells (4 × 10^6^) subcutaneously into the right rear of the mice.

### Pharmacokinetics

Sprague-Dawley rats (200-220 g) were used to investigate the pharmacokinetics of pCTX-S-CTX/PPa NPs (n = 5). PPa solution or pCTX-S-CTX/PPa NPs was intravenously injected at a PPa equivalent dose of 2 mg/kg. At predetermined intervals (0.083, 0.25, 0.5, 1, 2, 4, 8 and 12 h), blood was removed and centrifuged at 10,000 rpm for 5 min. The obtained plasma samples were stored at 20 ℃ for further analysis. PPa was extracted using a methanol protein precipitation method, and the concentration of PPa was measured using a microplate reader with excitation at 415 nm and emission collected at 675 nm.

### *Ex vivo* biodistribution

The biodistribution of pCTX-S-CTX/PPa NPs was studied in 4T1 tumor-bearing mice. When the tumor volume reached 300 mm^3^, the mice were randomly divided into six groups (n = 3) receiving PPa solution or pCTX-S-CTX/PPa NPs (2 mg/kg, PPa equivalent). At 4, 12 and 24 h, the mice were sacrificed and major organs were excised. Fluorescence signals from PPa in the tissues were imaged using an IVIS imaging system (PerkinElmer).

### *In vivo* chemo-photodynamic therapy

The therapeutic efficacy of pCTX-S-CTX/PPa NPs was studied in 4T1 tumor-bearing mice. When the tumor size reached ~150 mm^3^, the mice were randomly divided into seven groups (n = 5) receiving PBS, PPa solution + laser, CTX solution, PPa solution + CTX solution, CTX solution + PPa solution + laser, pCTX-S-CTX/PPa NPs, or pCTX-S-CTX/PPa NPs + laser. The formulations were administrated every other day (3 mg/kg for CTX and 3.8 mg/kg (two molar equivalents to CTX) for PPa. Laser irradiation (660 nm, 100 mW cm^-2^, 5 min) was administered to the laser-treated groups 12 h after injection for the pCTX-S-CTX/PPa NPs group or 4 h after injection for the PPa solution and the PPa solution + CTX solution groups. Tumor volumes and mouse body weights were recorded daily during treatment. On the third day after the last administration, the mice were sacrificed and blood samples were collected to obtain serum for analysis of liver and kidney functions. Heart, liver, spleen, lung, kidney and tumor tissues were harvested for H&E staining to observe physiological changes.

### Statistical analysis

Data are presented as mean ± standard deviation. Statistical differences were assessed using Student's t-test and one-way analysis of variance. P < 0.05 was considered a significant difference.

## Results and discussion

### Design and synthesis of a ROS-activable CTX homodimer

To construct a carrier-free co-delivery nanoplatform based on PPa-driven assembly of a homodimeric prodrug, a ROS-sensitive homodimer CTX prodrug (CTX-S-CTX) was synthesized via a single thioether bond. The synthetic route of CTX-S-CTX is shown in Figure S1. CTX was first coupled to TA to obtain an intermediate (CTX-TA) with a carboxyl group. Then, the CTX-S-CTX conjugate was synthesized by further coupling a CTX molecule with CTX-TA. The successful synthesis of CTX-S-CTX was confirmed by MS and ^1^H-NMR. As shown in Figure S2, the molecular ion peak was at m/z 1786.7, which was consistent with the predicted ionic formula [C_94_H_117_N_2_O_30_S+H]^+^. The MS result demonstrated that the actual molecular weight of CTX-S-CTX was correct. Moreover, we analyzed, ascribed and integrated each proton peak position in the ^1^H-NMR spectrum of CTX-S-CTX in detail, which was in accord with the molecular formula of CTX-S-CTX (Figure S3). All these results confirmed the successful synthesis of CTX-S-CTX.

### PPa-driven assembly of CTX-S-CTX

**Figure 2:**
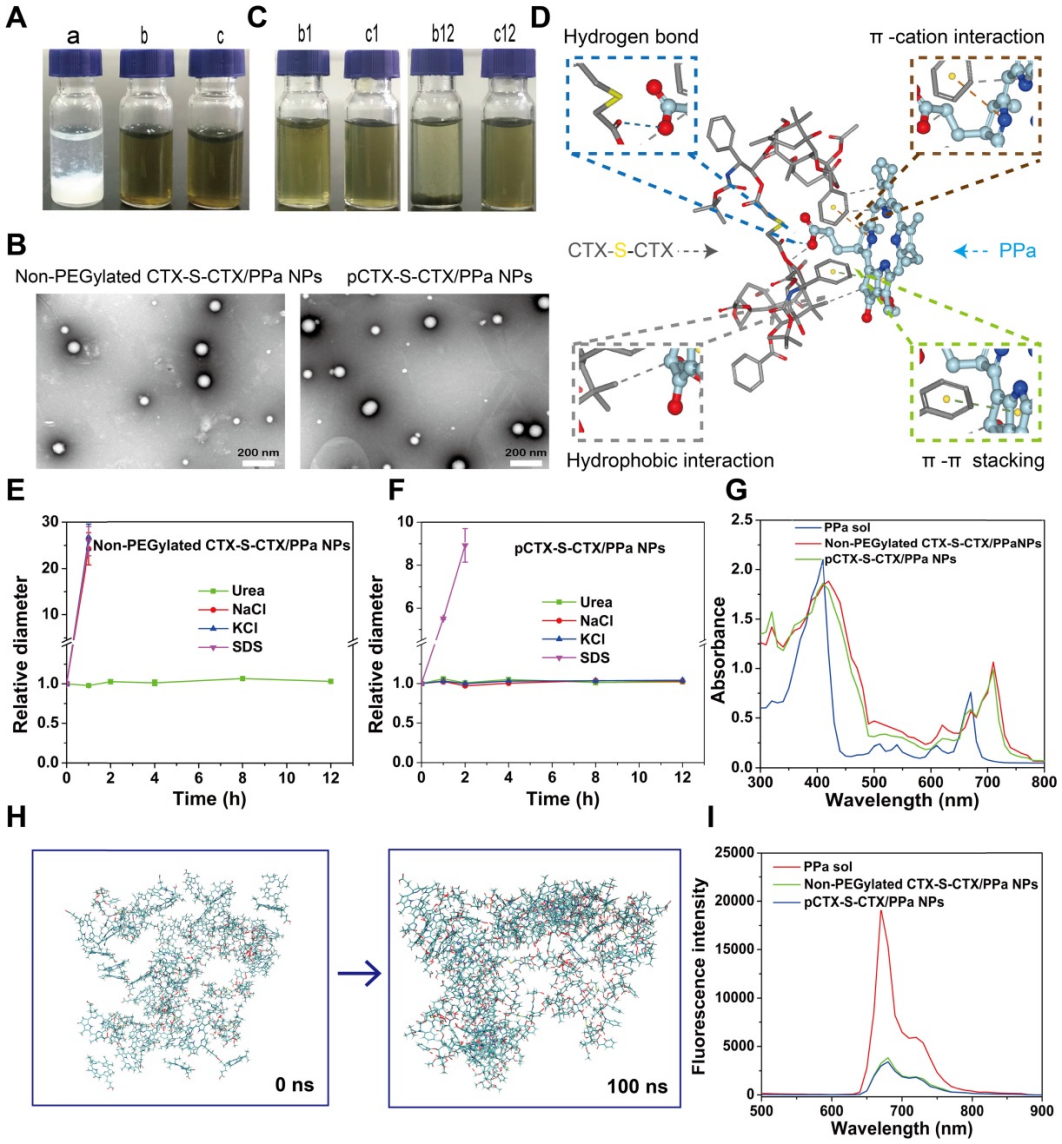
Nanoparticle properties of the PPa-driven CTX-S-CTX prodrug nanoassemblies. **(A)** Photographs of various formulations: a, precipitated CTX-S-CTX (1mg/mL); b, non-PEGylated CTX-S-CTX/PPa NPs; and c, pCTX-S-CTX/PPa NPs. **(B)** TEM images of non-PEGylated CTX-S-CTX/PPa NPs and PEGylated nanoassemblies (pCTX-S-CTX/PPa NPs).** (C)** Photographs of non-PEGylated CTX-S-CTX/PPa NPs (b) and pCTX-S-CTX/PPa NPs (c) incubated in PBS at 37 ℃ for 1 h (b1 and c1) or 12 h (b12 and c12).** (D)** Molecular docking result of CTX-S-CTX and PPa. **(E)** Changes in the particle size of non-PEGylated CTX-S-CTX/PPa NPs treated with urea (50 mM), NaCl (50 mM), KCl (50 mM), or SDS (50 mM). **(F)** Changes in the particle size of pCTX-S-CTX/PPa NPs treated with urea (50 mM), NaCl (50 mM), KCl (50 mM) and SDS (50 mM). **(G)** UV absorption spectra of PPa solution, non-PEGylated CTX-S-CTX/PPa NPs and pCTX-S-CTX/PPa NPs.** (H)** Molecular cluster changes of CTX-S-CTX and PPa (1:4) before and after simulation for 100 ns. **(I)** Fluorescence spectra of PPa solution, non-PEGylated CTX-S-CTX/PPa NPs, and pCTX-S-CTX/PPa NPs.

As previously mentioned, most homodimeric conjugates show very poor self-assembly capacities 25, and CTX-S-CTX was not an exception. When a solution of CTX-S-CTX dissolved in a mixture of ethyl alcohol and THF was added dropwise into water, it immediately aggregated and gradually precipitated (Figure 2A). As expected, the homodimeric conjugate of CTX was not able to self-assemble into NPs by itself. In comparison, CTX-S-CTX in the presence of PPa easily assembled into nanoassemblies (CTX-S-CTX/PPa NPs) in water by a nano-precipitation method (Figure 2A). To identify the optimal dose ratio of CTX-S-CTX and PPa, both the particle size and CI were used to screen formulations. When the dose ratio was set to 1:4 (CTX-S-CTX: PPa), both the particle size and the synergistic therapeutic effect were optimum (Table S1). Therefore, the following experiments were carried out using the nanoassemblies with the optimal dose ratio of 1:4. Non-PEGylated CTX-S-CTX/PPa NPs and PEGylated CTX-S-CTX/PPa NPs (pCTX-S-CTX/PPa NPs) were around 79 nm and 88 nm (Figure 2B) in size, with zeta potentials of approximately -15 and -20 mV, respectively (Table S2). PEGylation significantly improved the colloidal stability of the nanoassemblies by increasing resistance to salting-out. As shown in Figure 2C, the non-PEGylated CTX-S-CTX/PPa NPs gradually disintegrated and precipitated in PBS, while pCTX-S-CTX/PPa NPs showed good colloidal stability under the same conditions over 12 h. Moreover, compared to non-PEGylated CTX-S-CTX/PPa NPs, the particle size of pCTX-S-CTX/PPa NPs did not change significantly following incubation in PBS supplemented with 10% FBS at 37 °C for 12 h (Figure S4), suggesting that pCTX-S-CTX/PPa NPs had better stability in physiological medium.

PPa-driven assembly mechanisms were explored by molecular docking, intermolecular interaction destruction and molecular dynamics simulation 38-42. As shown in Figure 2D, multiple intermolecular interactions were involved in the assembly process, including hydrogen bond, hydrophobic interaction, π-π stacking interaction, and π-cation interaction. To further identify the main intermolecular forces involved, SDS, urea, NaCl, and KCl were utilized to break hydrophobic interaction, hydrogen bond interaction, electrostatic interaction and π-cation interaction in the nanoassemblies, respectively 39-40. As shown in Figure 2E-F, the particle sizes of non-PEGylated CTX-S-CTX/PPa NPs and pCTX-S-CTX/PPa NPs increased rapidly in the presence of SDS within 1 h, indicating the existence of hydrophobic interaction in the nanoassemblies. Moreover, non-PEGylated CTX-S-CTX/PPa NPs grew rapidly in NaCl and KCl solution, but pCTX-S-CTX/PPa NPs remained stable within 12 h. These results suggested that electrostatic interaction also played an important role in the assembly process of PPa and CTX-S-CTX, but PEGylation effectively protected the nanoassemblies from the influence of NaCl and KCl. Notably, there was no significant change in the particle sizes of both non-PEGylated CTX-S-CTX/PPa NPs and pCTX-S-CTX/PPa NPs in the presence of urea, suggesting that hydrogen bond was not a dominant force in the assembly process. Additionally, there was an obvious red shift in the UV absorption spectra of both non-PEGylated CTX-S-CTX/PPa NPs and pCTX-S-CTX/PPa NPs compared to PPa solution, suggesting the existence of π-π stacking interaction in the nanoassemblies (Figure 2G) 43, 44. The results from molecular dynamics simulation showed that PPa and CTX-S-CTX molecules immediately aggregated into clusters in aqueous solution within 100 ns (Figure 2H). As shown in Figure S5, the gyration radius of the aggregated molecules first decreased and then plateaued, which might be attributed to interactions between the molecules becoming much more intense and the intermolecular distance decreasing during the simulation. During the assembly process, obvious π-π stacking interactions were observed in the nanoassemblies (Figure S6). These results demonstrated that multiple interactions were involved in the assembly process of PPa and CTX-S-CTX and that hydrophobic interaction and π-π stacking interaction were the dominant forces. In comparison with PPa solution, both the non-PEGylated CTX-S-CTX/PPa NPs and pCTX-S-CTX/PPa NPs revealed reduced fluorescence intensity due to the ACQ effect of PPa in the nanoassemblies (Figure 2I).

### Effectively addressing the ACQ effect

**Figure 3:**
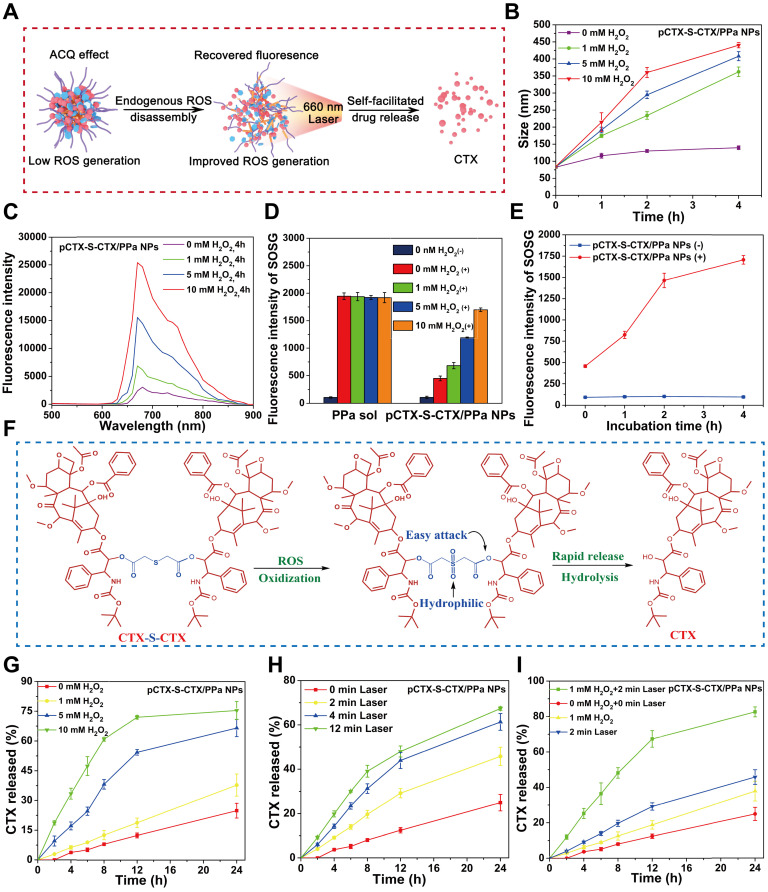
*In vitro* self-enhancing activation of CTX-S-CTX in pCTX-S-CTX/PPa NPs. **(A)** Schematic representation of the self-facilitated drug release and relief of PPa ACQ of pCTX-S-CTX/PPa NPs in the presence of endogenous ROS and laser irradiation.** (B)** Changes in the particle size of pCTX-S-CTX/PPa NPs in release media containing various concentrations of H_2_O_2_.** (C)** Changes in the fluorescence spectra of pCTX-S-CTX/PPa NPs incubated with various concentrations of H_2_O_2_. **(D)** ^1^O_2_ generation by PPa solution and pCTX-S-CTX/PPa NPs incubated with various concentrations of H_2_O_2_ under laser irradiation (660 nm, 100 mW/cm^2^, 5 min). (+): with laser, (-): without laser.** (E)** Singlet oxygen generation of PPa sol and pCTX-S-CTX/PPa NPs incubated with H_2_O_2_ (10 mM) under laser irradiation (660 nm, 100 mW/cm^2^, 5 min). (+): with laser, (-): without laser. **(F)** Schematic representation of drug release mechanism from CTX-S-CTX. **(G)** CTX release from pCTX-S-CTX/PPa NPs in release media containing various concentrations of H_2_O_2_.** (H)** CTX release from pCTX-S-CTX/PPa NPs in the media with various laser irradiation time (660 nm, 100 mW/cm^2^). **(I)** CTX release from pCTX-S-CTX/PPa NPs in release media containing H_2_O_2_ (1 mM) or/and with laser irradiation (660 nm, 100 mW/cm^2^, 2 min).

It is widely accepted that the fluorescence intensity of most PSs embedded in NPs substantially decreases due to the well-known ACQ effect, which also significantly impedes their photodynamic efficacy 23, 45. We hypothesized that the ACQ effect of PPa would be alleviated following disassembly of pCTX-S-CTX/PPa NPs triggered by endogenous ROS in tumor cells. The self-facilitated activation of CTX-S-CTX and disassembly of pCTX-S-CTX/PPa NPs in the presence of endogenous ROS is depicted in Figure 3A. First, the endogenous ROS is expected to break the single thioether bond in CTX-S-CTX, resulting in activation of most dimer prodrugs and disassembly of pCTX-S-CTX/PPa NPs. As shown in Figure 3B, the particle size of pCTX-S-CTX/PPa NPs incubated in release media containing various concentrations of H_2_O_2_ significantly increased over time, suggesting H_2_O_2_-triggered disassembly of pCTX-S-CTX/PPa NPs. As a result, the fluorescence intensity and ^1^O_2_ generation of PPa in pCTX-S-CTX/PPa NPs were obviously recovered in a H_2_O_2_ concentration- and incubation time-dependent manner (Figure 3C-E, Figure S7). All these results indicated that oxidation stimuli (*e.g.,* H_2_O_2_ and other ROS) successfully triggered the disassembly of pCTX-S-CTX/PPa NPs and effectively addressed the ACQ dilemma of PPa.

### Self-enhanced oxidation-sensitive prodrug activation

Selective and effective drug release from prodrug nanoassemblies in tumor cells is of crucial importance for high cancer treatment efficacy and low off-target toxicity 45, 46. The ROS-triggered activation process of CTX-S-CTX is shown in Figure 3F. The single thioester bond could be oxidized into hydrophilic sulfoxide groups, significantly increasing the hydrophilicity of CTX-S-CTX. This increased hydrophilicity could facilitate rapid release of CTX from CTX-S-CTX (Figure 3G-I). The release profile of CTX from pCTX-S-CTX/PPa NPs was investigated. As shown in Figure 3G, less than 20% of CTX was released within 24 h in release medium, while more than 70% of CTX was released within 12 h in release medium containing 10 mM H_2_O_2_. Furthermore, pCTX-S-CTX/PPa NPs showed a laser-triggered drug release in release medium due to ROS production by PPa. As shown in Figure 3H, more than 60% of CTX was released from pCTX-S-CTX/PPa NPs within 24 h in release medium following laser irradiation (660 nm, 100 mW/cm^2^, 12 min). Notably, more than 80% of CTX was released from pCTX-S-CTX/PPa NPs in the presence of H_2_O_2_ (1 mM) within 24 h following laser irradiation (660 nm, 100 mW/cm^2^, 2 min) (Figure 3I), indicating the self-enhanced oxidation-sensitive prodrug activation and release features of the PPa-driven nanoassemblies.

### Cellular uptake

**Figure 4:**
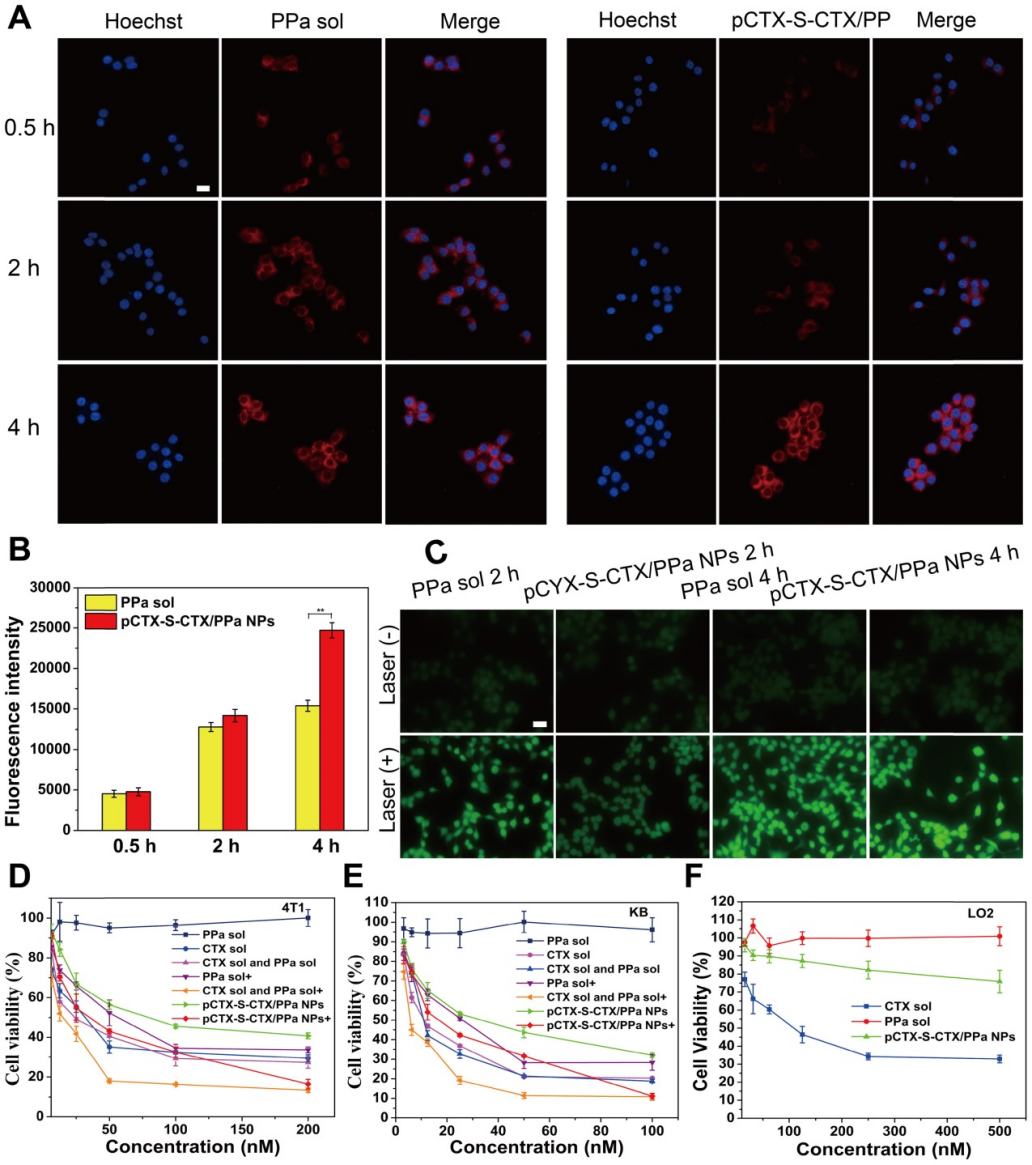
*In vitro* cellular evaluation of pCTX-S-CTX/PPa NPs (n = 3). **(A)** CLSM images of 4T1 cells incubated with PPa solution or pCTX-S-CTX/PPa NPs for 0.5, 2, or 4 h. **(B)** Fluorescence intensity of PPa in 4T1 cells incubated with PPa solution or pCTX-S-CTX/PPa NPs for 0.5, 2, or 4 h. **(C)** Cellular ROS production in 4T1 cells stained with DCFH-DA after incubation with PPa solution or pCTX-S-CTX/PPa NPs for 2 or 4 h with/without laser irradiation. **(D-F)** Cell viability of 4T1, KB and LO2 cells treated with various formulations. * P < 0.05, ** P < 0.01, and *** P < 0.005. (+): with laser, (-): without laser. Scale bar = 10 μm.

The cellular uptake efficiency of pCTX-S-CTX/PPa NPs was initially evaluated by observing the intracellular fluorescence of PPa. As shown in Figure 4A, cells treated with pCTX-S-CTX/PPa NPs showed much weaker fluorescence than cells treated with PPa solution at both 0.5 and 2 h. However, the two groups showed comparable fluorescence intensities after longer incubation (4 h). The weak fluorescence of cells incubated with pCTX-S-CTX/PPa NPs for short periods should be attributed to the ACQ effect. With extension of the incubation time, the ACQ effect was significantly relieved due to the ROS-triggered disassembly of pCTX-S-CTX/PPa NPs, resulting in enhanced intracellular fluorescence. To eliminate the interference of ACQ effect and accurately evaluate the cellular uptake of pCTX-S-CTX/PPa NPs, an ultrasonic method was used to break the cells and extract intracellular PPa. As shown in Figure 4B, the cellular uptake of pCTX-S-CTX/PPa NPs was time-dependent, and the concentration of PPa in cells treated with pCTX-S-CTX/PPa NPs was obviously higher than that of cells treated with PPa solution at 4 h. Most free drugs enter cells by passive diffusion in a concentration-dependent manner 47. By contrast, the cellular uptake of nanomedicines has been found to be realized by endocytosis independent of concentration gradients, which usually enables higher cellular uptake efficiency than free drug solutions 48. In particular, PPa is a polar molecule with a large molecular weight and poor water-solubility. As a result, it is not easy for PPa to move across cell membranes. Thus, pCTX-S-CTX/PPa NPs exhibited higher cellular uptake efficiency than PPa solution.

### Cellular ROS generation

Cellular ROS generation was detected using the probe DCFH-DA. As shown in Figure 4C, cells incubated with pCTX-S-CTX/PPa NPs for 2 h and then exposed to laser irradiation exhibited weaker probe fluorescence than cells treated with solution. This inefficient ROS generation by the NPs should be attributed to the ACQ effect of PPa. Notably, the fluorescence signal from DCF in cells that were treated with pCTX-S-CTX/PPa NPs for 4 h was significantly enhanced, indicating that ACQ of PPa was alleviated over time. These results confirmed that the nanostructure of pCTX-S-CTX/PPa NPs could be gradually disassembled in the presence of intracellular ROS. As a result, the ACQ effect of PPa in NPs was significantly alleviated with extension of the incubation time, leading to a sharp increase in ROS production.

### Synergistic cytotoxicity

As discussed above, the CIs of CTX solution and PPa solution in various ratios were evaluated in 4T1 cells (Table S1). When the dose ratio of CTX solution and PPa solution was 1:2, the optimal synergistic cytotoxicity (0.45) was obtained. We further investigated the cytotoxicity of pCTX-S-CTX/PPa NPs against 4T1 cells and KB cells. As shown in Figure 4D-E, Figure S8-S9, PPa solution showed almost no cytotoxicity to tumor cells without laser irradiation. The mixture of CTX and PPa exhibited a comparable cytotoxicity as CTX solution without laser irradiation. pCTX-S-CTX/PPa NPs exhibited weaker cytotoxicity than CTX solution without laser irradiation. Under laser irradiation, pCTX-S-CTX/PPa NPs showed more potent cytotoxicity with laser irradiation than without. Moreover, pCTX-S-CTX/PPa NPs demonstrated comparable synergistic cytotoxicity to the mixed solution of CTX and PPa with laser irradiation. The result should be attributed to the high cellular uptake, relieved ACQ effect of PPa, and self-enhancing drug release of pCTX-S-CTX/PPa NPs. In addition, LO2 cells were used to evaluate the therapeutic selectivity of the ROS-responsive pCTX-S-CTX/PPa NPs. As shown in Figure 4F, CTX solution still showed potent cytotoxicity to LO2 cells under dark conditions. By contrast, the ROS-responsive pCTX-S-CTX/PPa NPs exhibited negligible cytotoxicity to LO2 cells under the same condition, due to the low intracellular ROS concentration in LO2 cells. The IC_50_ values of pCTX-S-CTX/PPa NPs in 4T1, KB, and LO2 cells were calculated and are shown in Table S3-4. These results demonstrated that pCTX-S-CTX/PPa NPs could not only perform potent chemo-photodynamic therapy under laser irradiation, but also effectively reduce the systemic toxicity of CTX.

### Pharmacokinetics and biodistribution

**Figure 5:**
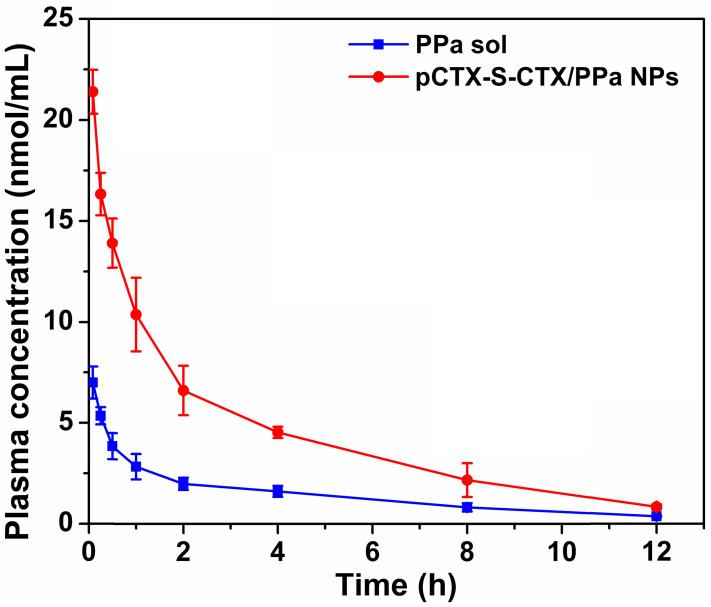
Plasma concentration-time profiles of PPa solution and pCTX-S-CTX/PPa NPs (n = 5).

The pharmacokinetic profiles of PPa solution and pCTX-S-CTX/PPa NPs were measured using the fluorescence emission of PPa. The pharmacokinetic parameters were calculated and are shown in Table S5. As shown in Figure 5, pCTX-S-CTX/PPa NPs significantly prolonged the circulation time in blood compared with PPa solution, with a threefold increase in the area under the concentration time curve (AUC).

**Figure 6:**
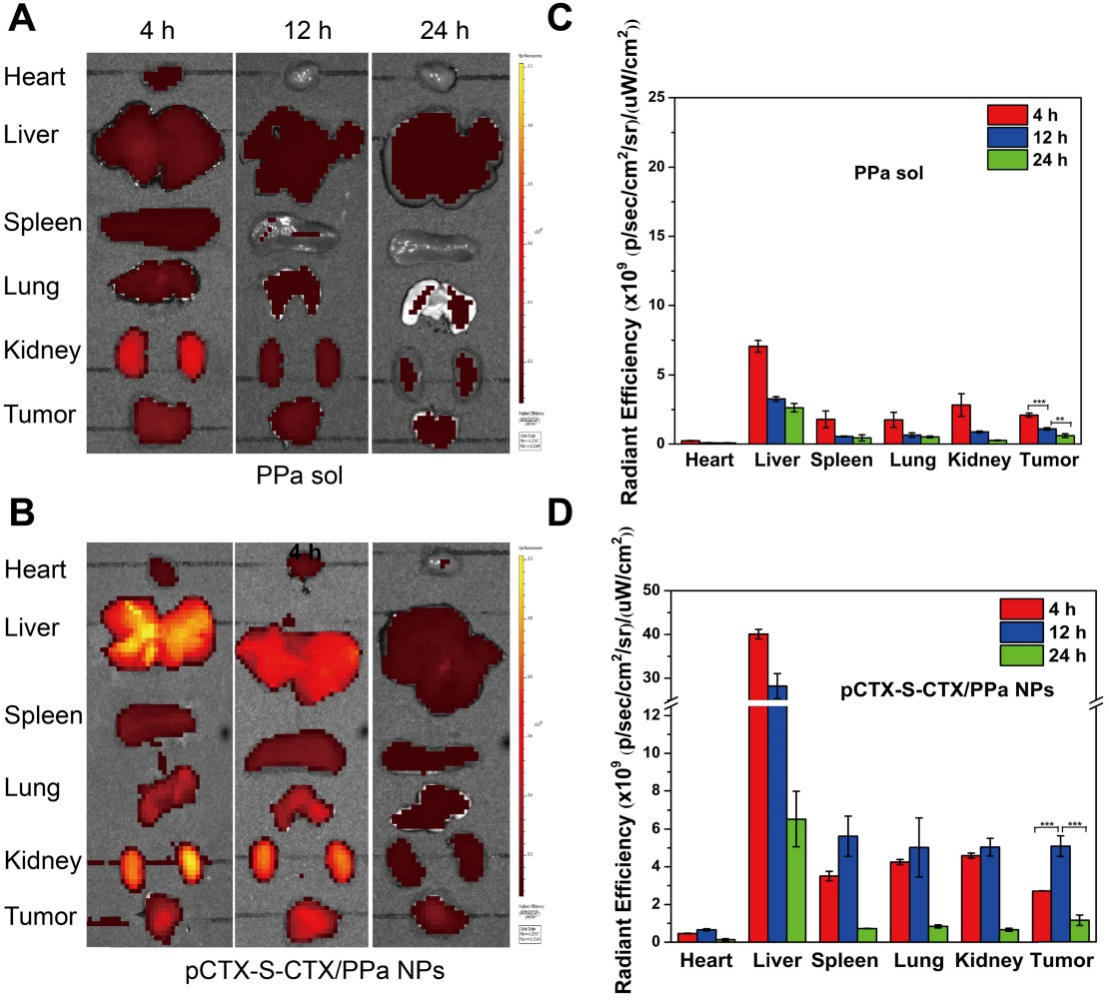
*Ex vivo* biodistribution of pCTX-S-CTX/PPa NPs in 4T1 tumor-bearing mice (n = 3).** (A)** Fluorescent images of major organs and tumors 4, 12 and 24 h after injection of PPa solution.** (B)** Fluorescence images of major organs and tumors 4, 12 and 24 h after injection of pCTX-S-CTX/PPa NPs. **(C)** Quantitative analysis of A.** (D)** Quantitative analysis of B. * P < 0.05, ** P < 0.01, and *** P < 0.005.

The *ex vivo* biodistribution of pCTX-S-CTX/PPa NPs was evaluated in 4T1 tumor-bearing BALB/c mice using the near-infrared fluorescence from PPa. As shown in Figure 6A-D, free PPa was quickly eliminated from the body and showed low accumulation in tumors. In contrast, pCTX-S-CTX/PPa NPs efficiently accumulated in tumors, with the strongest fluorescence intensity observed at 12 h. The result should be attributed to the good stability and long circulation time of pCTX-S-CTX/PPa NPs, which enabled tumor accumulation by the enhanced permeability and retention effect.

### *In vivo* synergistic chemo-photodynamic therapy

**Figure 7:**
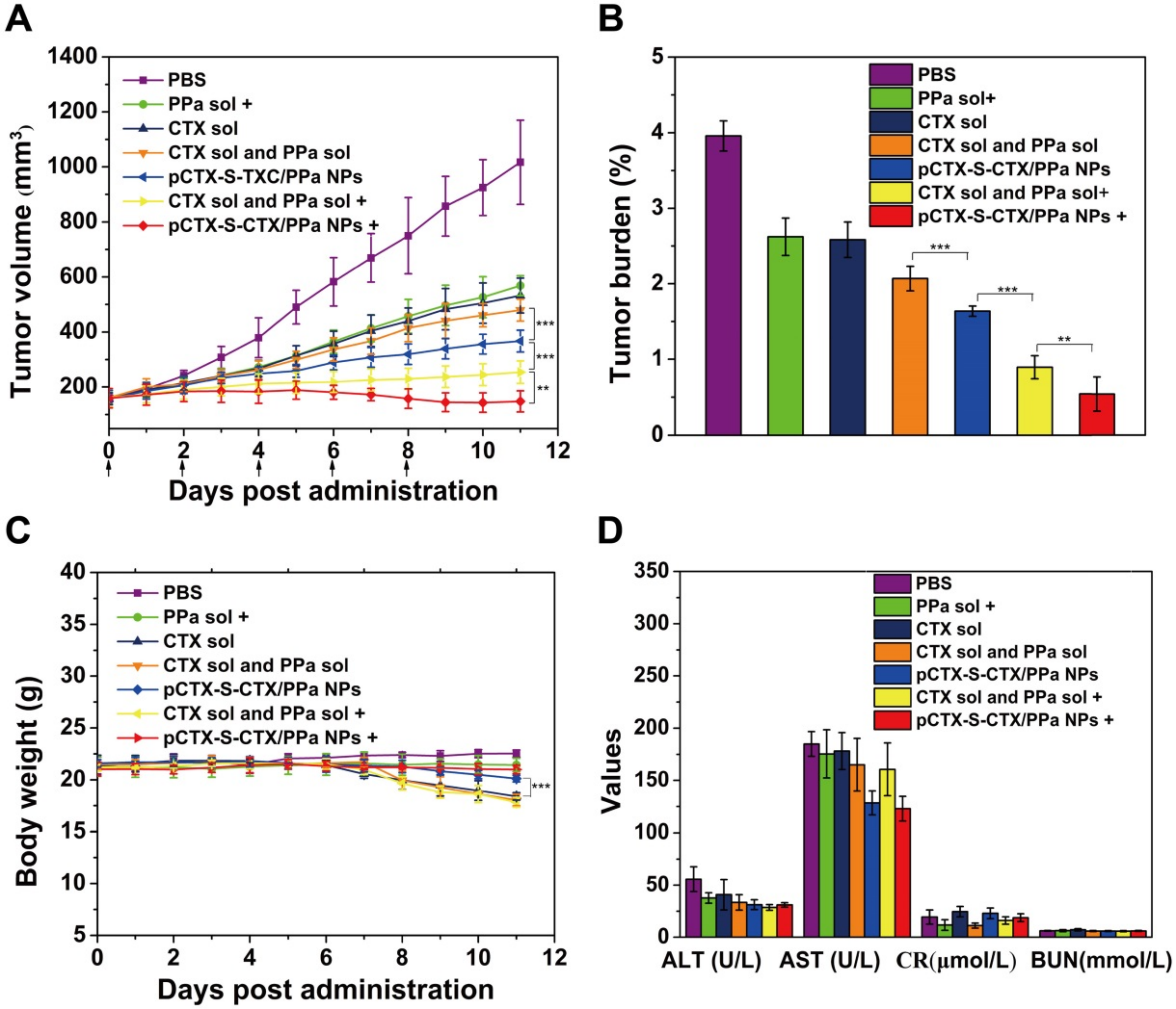
*In vivo* therapeutic efficacy of pCTX-S-CTX/PPa NPs in 4T1 tumor-bearing mice (n = 5).** (A)** Tumor volume growth curves after various treatments. **(B)** Tumor burden after the last treatment calculated as the ratio of tumor and mouse weights. **(C)** Body weight changes of mice during treatment.** (D)** Liver and kidney functional parameters after the last treatment. * P < 0.05, ** P < 0.01 and *** P < 0.005. (+): with laser, (-): without laser.

The *in vivo* antitumor activity of pCTX-S-CTX/PPa NPs was studied in 4T1 tumor-bearing BALB/c mice. As shown in Figure 7A, rapid tumor growth was observed in mice treated with PBS. By contrast, the other treatments suppressed tumor growth with varying efficiencies (Figure 7A-B, Figure S10). Among them, CTX solution and the mixed of CTX and PPa without laser irradiation exhibited comparable antitumor activity to PPa solution with laser irradiation. pCTX-S-CTX/PPa NPs without laser suppressed tumor growth, with the tumor volume only growing to 300 mm^3^ by the 15th day after the first administration. Compared to PPa solution alone, both pCTX-S-CTX/PPa NPs and the mixture of CTX and PPa more significantly suppressed tumor growth with laser irradiation. Moreover, pCTX-S-CTX/PPa NPs more significantly suppressed tumor growth than the mixture of CTX and PPa with laser irradiation. The potent synergistic chemo-photodynamic therapy provided by pCTX-S-CTX/PPa NPs should be attributed to its multiple therapeutic advantages, including high drug loading, good colloidal stability, long circulation time, high tumor accumulation, efficient cellular uptake, relieved ACQ effect of PPa and self-enhanced ROS-responsive drug release.

Additionally, pCTX-S-CTX/PPa NPs revealed good safety during treatment. As shown in Figure 7C, there was no noticeable change in the body weights of mice treated with any treatment except for CTX solution and the mixture of CTX and PPa with and without laser irradiation. Also, no significant abnormality in hepatorenal function was found (Figure 7D). Moreover, no obvious damage to the major organs was observed by H&E staining (Figure S11). Notably, distinct necrotic and apoptotic regions were observed in tumor tissues treated with pCTX-S-CTX/PPa NPs with laser irradiation, suggesting the potency of the synergistic chemo-photodynamic therapy.

## Conclusions

In summary, carrier-free PPa-driven nanoassemblies of homodimeric prodrug were rationally designed for self-enhancing prodrug activation and synergistic chemo-photodynamic therapy. We synthesized a novel homodimeric prodrug composed of CTX bridged by a single thioether bond. We found that PPa was able to drive the assembly of CTX-S-CTX into stable NPs. More importantly, the dose ratio of PPa and CTX could be freely adjusted in the nanoassemblies to achieve synergistic therapeutic effects. The nanostructure of the PPa-driven nanoassemblies was readily disassembled in the presence of endogenous ROS, efficiently addressing the ACQ dilemma of PPa. Furthermore, ROS generated by PPa under laser irradiation together with the endogenous ROS synergistically promoted rapid release of CTX from CTX-S-CTX. As a result, pCTX-S-CTX/PPa NPs showed potent chemo-photodynamic antitumor activity *in vivo*. This study offers a simple and effective strategy to facilitate the self-assembly of homodimeric prodrugs, and provides a novel co-delivery modality for chemo-photodynamic antitumor therapy.

## Supplementary Material

Supplementary figures and tables.
