# Supplementary material for: Photosensitizer-driven nanoassemblies of homodimeric prodrug for self-enhancing activation and synergistic chemo-photodynamic therapy
Source: Theranostics. 2021 Apr 7;11(12):6019–32. doi: 10.7150/thno.59065 (PMC8058734; doi:10.7150/thno.59065)
Supplement: Supplementary file 1 — Supplementary figures and tables. [file thnov11p6019s1.pdf]

Supplementary Material

Photosensitizer-driven nanoassemblies of homodimeric prodrug for self-enhancing activation and synergistic chemo-photodynamic therapy

Authors: Shenwu Zhang[a], Ziyue Wang[a], Zhiqiang Kong[a], Yuequan Wang[a], Xuanbo Zhang[a], Bingjun Sun[a], Haotian Zhang[b], Qiming Kan[b], Zhonggui He, Cong Luo[a,*] and Jin Sun[a,*]

Affiliations:

[a]Department of Pharmaceutics, Wuya College of Innovation, Shenyang Pharmaceutical University, Shenyang, Liaoning, 110016, P. R. China

[b]Department of Pharmacology, School of Life Science and Biopharmaceutics, Shenyang Pharmaceutical University, Shenyang, Liaoning 110016, PR China

***Corresponding authors:**

Cong Luo, Ph.D. and Jin Sun, Ph.D.

Department of Pharmaceutics, Wuya College of Innovation, Shenyang Pharmaceutical University, Shenyang, Liaoning, 110016, P. R. China

Tel: +86-24-23986321; Fax: +86-24-23986321

E-mail: luocong@syphu.edu.cn; sunjin@syphu.edu.cn

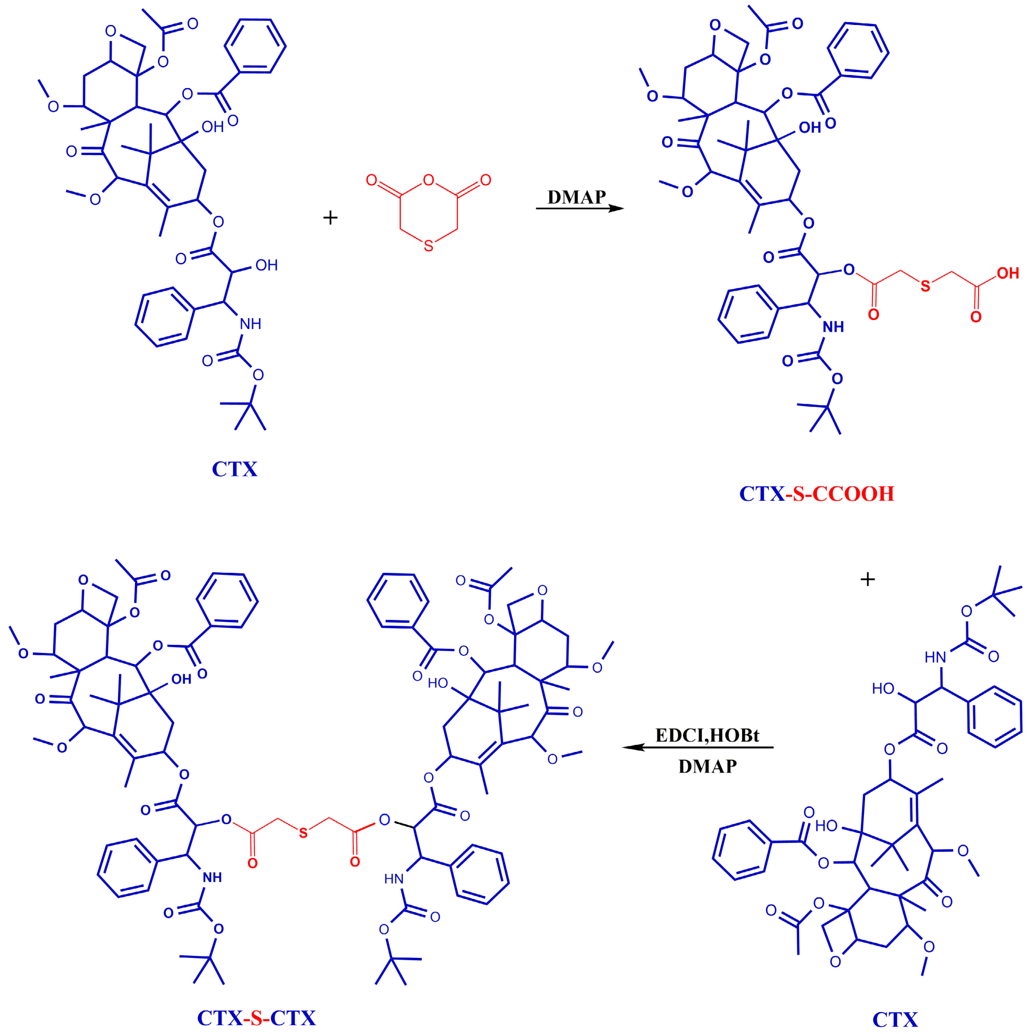

Figure S1. Synthetic route of CTX-S-CTX.

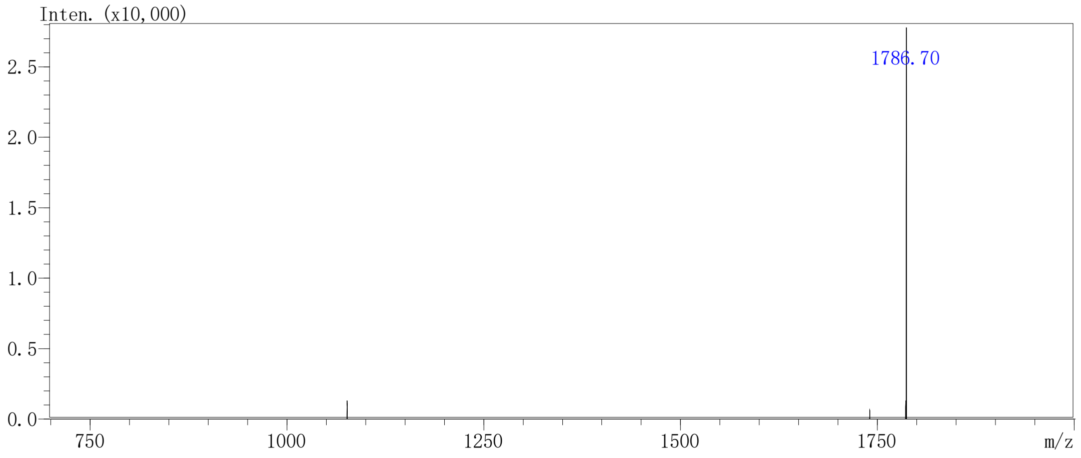

Figure S2. Mass spectrum of CTX-S-CTX.

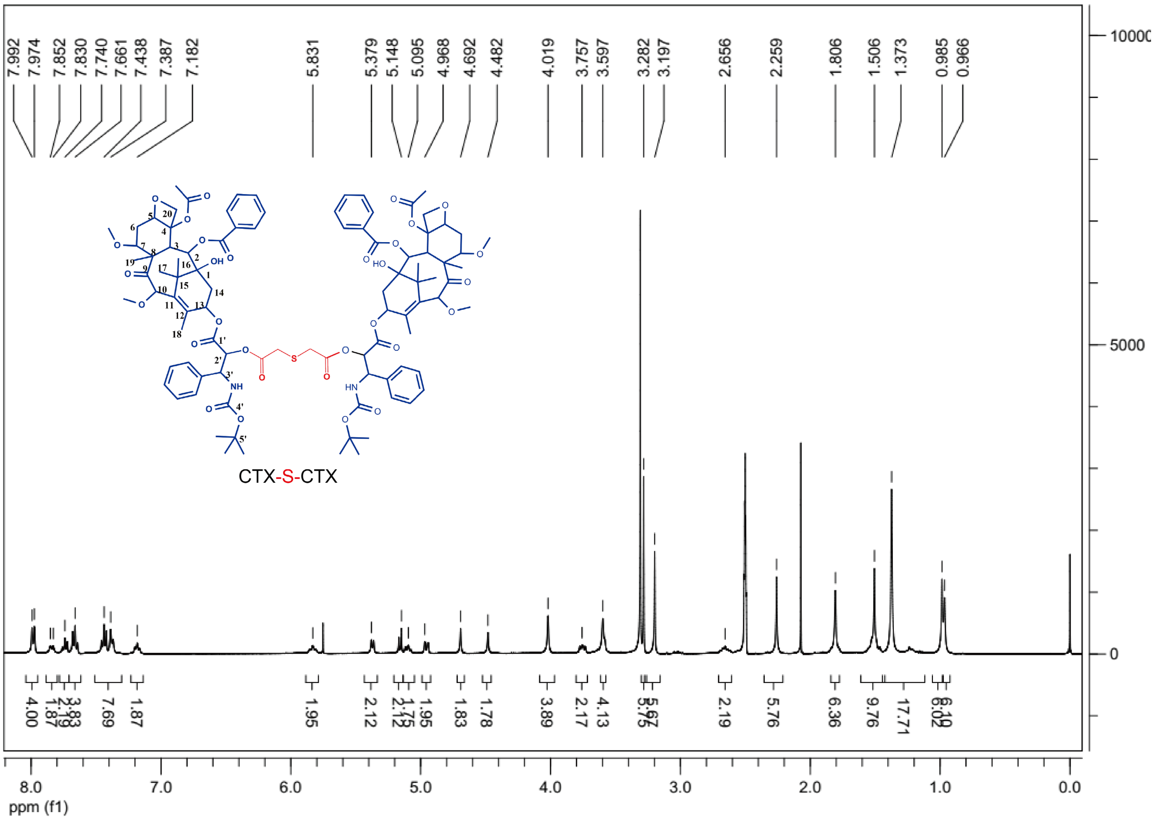

Figure S3. ^{1}H-NMR spectrum of CTX-S-CTX

^{1}H NMR results: ^{1}H NMR (400 MHz, DMSO-d6, ppm): δ 7.97-7.99 (d, 4H, Ar-H), 7.83-7.85 (d, 2H, Ar-H), 7.740 (t, 2H, Ar-H), 7.661 (t, 4H, Ar-H), 7.39-7.44 (d, 6H, Ar-H), 7.182 (t, 2H, –NH–), 5.831 (t, 2H, 13-CH), 5.379 (d, 2H, 2-CH), 5.148 (d, 2H, 3'-H), 5.095 (t, 2H, 10-CH),4.968 (s, 2H, 2'-H), 4.692 (d, 2H, 5-CH), 4.482 (d, 2H, 7-H), 4.019 (s, 4H, 20-CH_2), 3.757 (m, 2H, 7-CH), 3,597 (s, 4H, CH_2SCH_2), 3.282 (s, 6H, 10-OCH_3), 3.197 (s, 6H, 7-OCH_3), 2.656 (m, 2H, 3-CH), 2.259 (s, 6H, -OAc), 1.806 (s, 6H, 18-CH_3), 1.506 (10H, 14-CH_2, 19-CH_3), 1.668 (s, 6H, 19-CH_3), 1.373 (s, 18H, $C(CH_3)_3$), 0.985 (s, 6H, 16-CH_3), 0.966 (s, 6H, 17-CH_3).

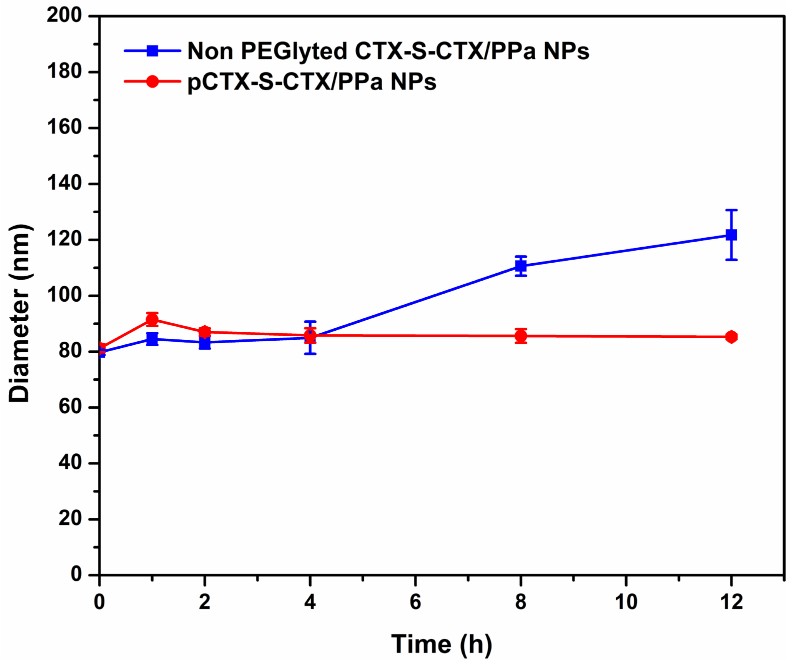

Figure S4. Changes in particle size of non-PEGylated CTX-S-CTX/PPa NPa and pCTX-S-CTX/PPa NPs in PBS containg 10% FBS.

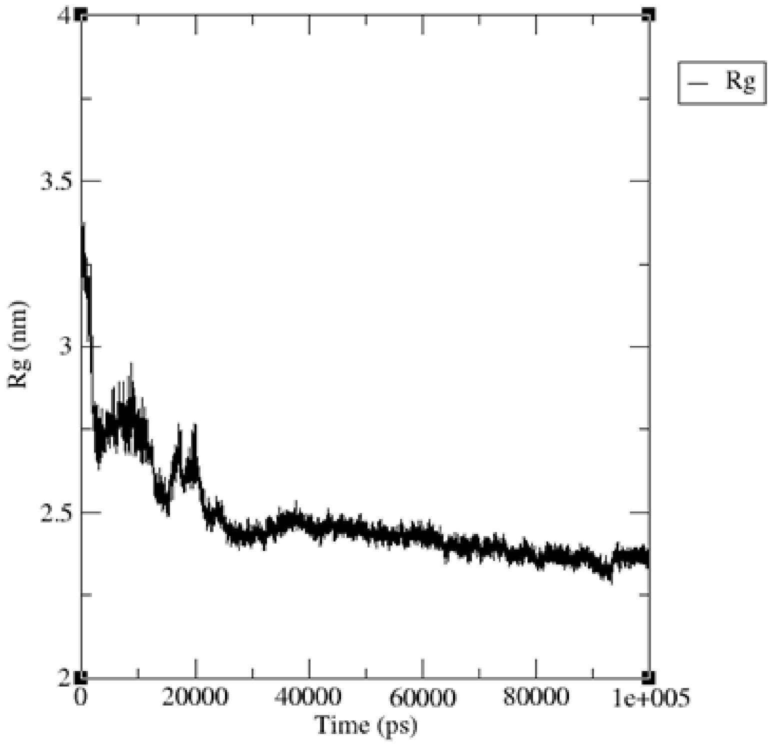

Figure S5. Gyration radius of the aggregated molecules during simulation.

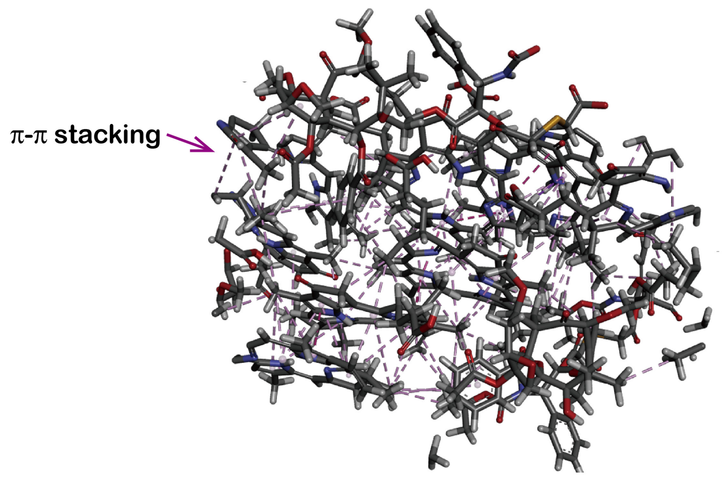

Figure S6. π-π stacking interaction between the selected PPa and other molecules within 1 nm after assembly.

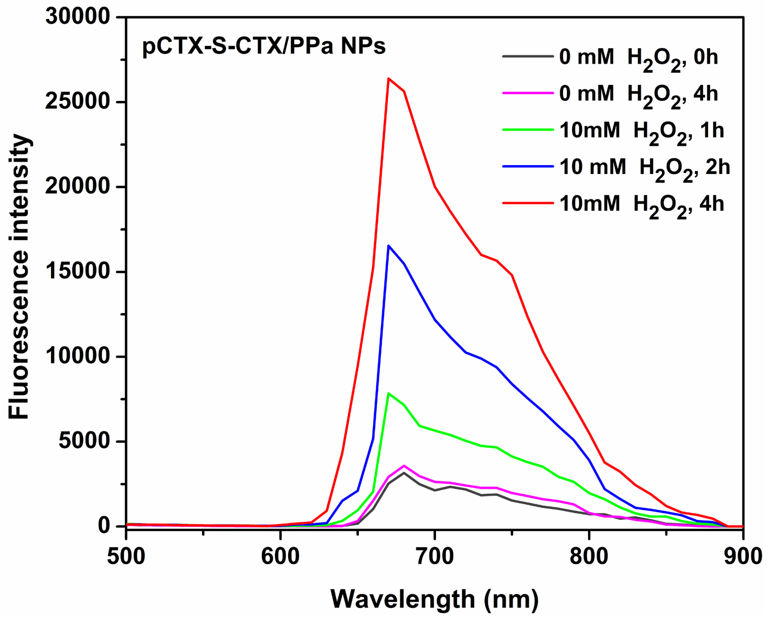

Figure S7. Fluorescence intensity of pCTX-S-CTX/PPa NPs in release medium containing H_2O_2 (10 mM) for 1, 2, or 4 h.

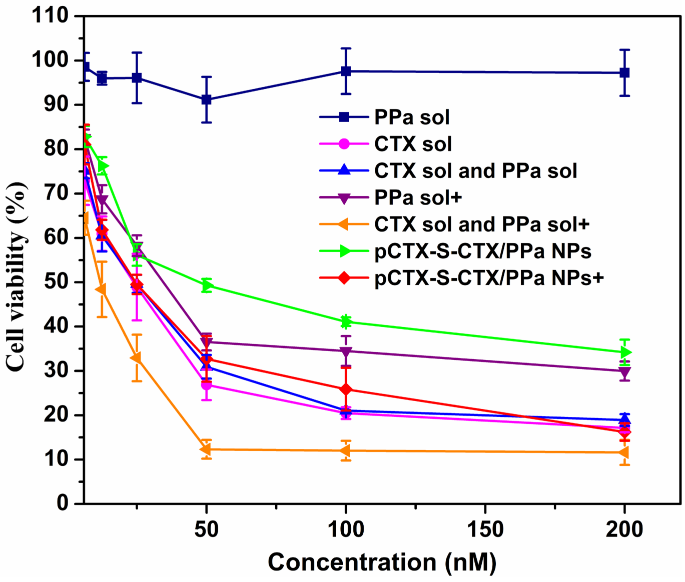

Figure S8. Cell viability of 4T1 cells treated with various formulations for 72 h. (+): with laser, (-): without laser.

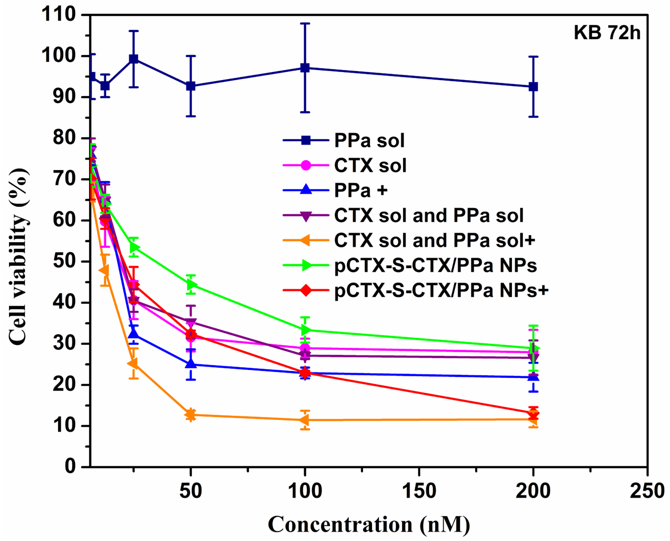

Figure S9. Cell viability of KB cells treated with various formulations for 72 h. (+): with laser, (-): without laser.

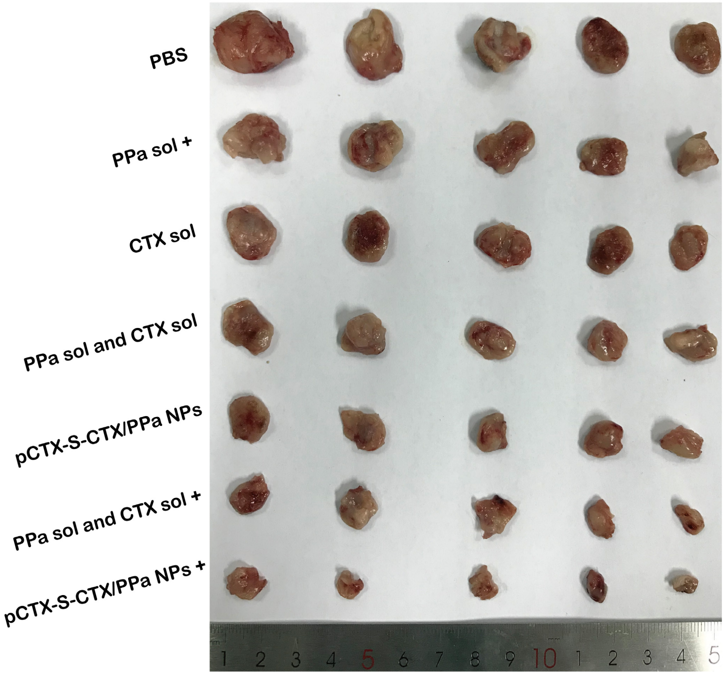

Figure S10. Photographs of excised tumor after various treatments. (+): with laser, (-): without laser.

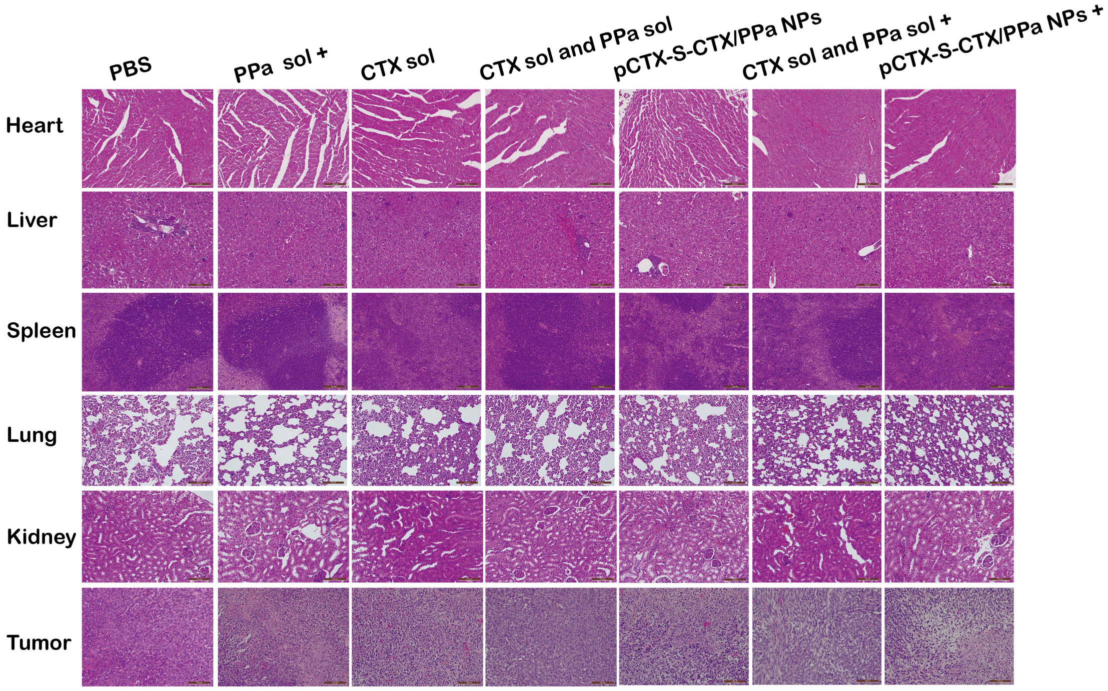

Figure S11. H&E staining of major organs and tumors. Scale bar = 100 μm. (+): with laser, (-): without laser.

Table S1. Characterization of non-PEGylated CTX-S-CTX/PPa NPs and combination index (CI) of CTX sol and PPa sol (+) at various ratios, (+): with laser (n=3).

CTX-S-CTX/PPa	Diameter (nm)	PDI	CTX/PPa	CI (CTX and PPa)
1:8	90.45±3.50	0.22±0.027	1:4	0.87
1:6	88.74±1.76	0.19±0.02	1:3	0.53
1:4	79.28±3.91	0.17±0.04	1:2	0.45
1:2	77.71±1.38	0.16±0.10	1:1	0.91
1:1	76.64±1.67	0.15±0.03	2:1	1.01
3:2	78.27±3.51	0.205±0.07	3:1	1.03
2:1	79.22±1.81	0.19±0.08	4:1	1.07

Notes: $CI = X \times AB_{\text{combination}}/A_{\text{alone}} + Y \times AB_{\text{combination}}/B_{\text{alone}}$. X is the dose proportion of CTX in the mixture solution of PPa and CTX, Y is the dose proportion of PPa in the mixture solution of PPa and CTX, A_{alone} is the IC_{50} of CTX solution, B_{alone} is the IC_{50} of PPa solution, and $AB_{combiantion}$ is the IC_{50} of mixture solution.

Table S2 Characterization of CTX-S-CTX/PPa NPs and the CIs of CTX solution and PPa solution with laser irradiation (+) at various ratios (n = 3).

Formulations	Diameter (nm)	PDI	Zeta (mV)	Drug loading
Non-PEGylated CTX-S-CTX/PPa NPs	79.28±3.91	0.17±0.04	-14.6±0.56	97.1%
pCTX-S-CTX/PPa NPs	88.70±3.08	0.19±0.03	-19.9±1.40	80.9%

Table S3 IC_{50} values of various formulations against 4T1 or KB cells at 48 or 72 h (n = 3). (+): with laser, (-): without laser.

Formulations	4T1		KB	
	48 h	72 h	48 h	48 h
PPa sol (-)	>1000	>1000	>1000	>1000
CTX sol (-)	31.59±3.54	20.34±0.83	13.82±0.26	9.30±0.66
PPa sol (+)	57.35±3.24	41.10±1.14	23.59±3.06	18.95±1.02
CTX sol and PPa sol (-)	34.93±4.16	22.73±1.72	12.89±2.07	10.05±0.76
CTX sol and PPa sol (+)	14.56±1.40	10.30±0.67	6.97±0.34	4.93±0.71
pCTX-S-CTX/PPa NPs (-)	87.17±3.92	55.96±4.29	33.59±1.57	27.30±3.76
pCTX-S-CTX/PPa NPs (+)	39.77±1.59	26.54±1.27	16.86±1.99	10.70±0.14

Table S4. IC_{50} values of PPa solution (-), CTX sol (-), and pCTX-S-CTX/PPa NPs (-) against 4T1 cells at 48 h (n = 3). (+): with laser, (-): without laser.

Formulations	LO2
PPa sol (-)	>1000
CTX sol (-)	111.18±3.51
pCTX-S-CTX/PPa NPs (-)	>1000

Table S5. Pharmacokinetic parameters of PPa solution and pCTX-S-CTX/PPa NPs (n = 5).

Formulations	$AUC_{(0-\infty)}$（μg/L*h）	$t_{1/2}$（h）	C_{max}（μg /L）
PPa sol	9453.82±1437.54	3.67±0.41	3732.95±426.33
pCTX-S-CTX/PPa NPs	28761.59±1098.95	3.37±0.51	11425.56±578.82
